# Deficiency in the production of antibodies to lipids correlates with increased lipid metabolism in severe COVID-19 patients

**DOI:** 10.3389/fimmu.2023.1188786

**Published:** 2023-06-23

**Authors:** Ignacio Piédrola, Sara Martínez, Ana Gradillas, Alma Villaseñor, Vanesa Alonso-Herranz, Isabel Sánchez-Vera, Esther Escudero, Isabel A. Martín-Antoniano, Jose Felipe Varona, Andrés Ruiz, Jose María Castellano, Úrsula Muñoz, María C. Sádaba

**Affiliations:** ^1^ Instituto de Medicina Molecular Aplicada (IMMA), Facultad de Medicina, Universidad San Pablo-CEU, CEU Universities, Boadilla del Monte, Madrid, Spain; ^2^ Centro de Metabolómica y Bioanálisis (CEMBIO), Facultad de Farmacia, Universidad San Pablo-CEU, CEU Universities, Boadilla del Monte, Madrid, Spain; ^3^ Genetic and Molecular Epidemiology Group, Spanish National Cancer Research Centre (CNIO), Madrid, Spain; ^4^ Servicio de Medicina Interna, Hospital Universitario Hospitales de Madrid (HM), Boadilla del Monte, Madrid, Spain

**Keywords:** natural antibodies, COVID-19, IgM, inflammation, lipidomic, lysophosphatidylcholine, lysophosphatidylethanolamine, phosphatidylinositol

## Abstract

**Background:**

Antibodies to lipids are part of the first line of defense against microorganisms and regulate the pro/anti-inflammatory balance. Viruses modulate cellular lipid metabolism to enhance their replication, and some of these metabolites are proinflammatory. We hypothesized that antibodies to lipids would play a main role of in the defense against SARS-CoV-2 and thus, they would also avoid the hyperinflammation, a main problem in severe condition patients.

**Methods:**

Serum samples from COVID-19 patients with mild and severe course, and control group were included. IgG and IgM to different glycerophospholipids and sphingolipids were analyzed using a high-sensitive ELISA developed in our laboratory. A lipidomic approach for studying lipid metabolism was performed using ultra-high performance liquid chromatography coupled to electrospray ionization and quadrupole time-of-flight mass spectrometry (UHPLC-ESI-QTOF-MS).

**Results:**

Mild and severe COVID-19 patients had higher levels of IgM to glycerophosphocholines than control group. Mild COVID-19 patients showed higher levels of IgM to glycerophosphoinositol, glycerophosphoserine and sulfatides than control group and mild cases. 82.5% of mild COVID-19 patients showed IgM to glycerophosphoinositol or glycerophosphocholines plus sulfatides or glycerophosphoserines. Only 35% of severe cases and 27.5% of control group were positive for IgM to these lipids. Lipidomic analysis identify a total of 196 lipids, including 172 glycerophospholipids and 24 sphingomyelins. Increased levels of lipid subclasses belonging to lysoglycerophospholipids, ether and/or vinyl-ether-linked glycerophospholipids, and sphingomyelins were observed in severe COVID-19 patients, when compared with those of mild cases and control group.

**Conclusion:**

Antibodies to lipids are essential for defense against SARS-CoV-2. Patients with low levels of anti-lipid antibodies have an elevated inflammatory response mediated by lysoglycerophospholipids. These findings provide novel prognostic biomarkers and therapeutic targets.

## 1 Introduction

The course of COVID-19, a disease caused by SARS-CoV-2 infection, is heterogeneous. More than 40% of COVID-19 patients are thought to be asymptomatic (1, 2), but others develop the disease in the following severity categories: mild, severe, and critical. Mild cases may or may not suffer from pneumonia, whereas severe cases show dyspnea and hypoxia, and critical cases suffer from severe pneumonia, cardiac arrest, and multiple organ failure (2–4).

Viruses, including SARS-CoV-2, consist of genetic material packaged in the capsid, which is mainly composed of lipids (5). Antibodies to lipids are mainly IgM (6) and are the first line of defense against viruses, such as influenza (7–15), lymphocytic choriomeningitis (16), vesicular stomatitis (16), and human immunodeficiency virus (HIV) infection (17–19). Natural antibodies also regulate the pro/anti-inflammatory balance (20). In this context, the Coronaviridae family hijacks the lipid metabolism to induce the production of essential viral membrane lipids, such as lysoglycerophospholipids (LysoGPs) and arachidonic acid (21, 22). These molecules are pro-inflammatory and promote the recruitment of monocytes, the numbers of which are increased in the lungs of patients with severe COVID-19 (23, 24).

Based on these data, we hypothesized that antibodies to lipids might play a main role in the defense against SARS-CoV-2 virus and that the deficit of this humoral immune response could lead to a proinflammatory lipid profile. Therefore, we aimed to analyze the presence of serum IgG and IgM anti-lipid antibodies using the most sensitive assay (25, 26) (patent ES2768783) to clarify the role of these antibodies in COVID-19 patients.

In addition, to investigate the relationship of antibodies to lipids and inflammation, a semi-targeted lipidomic analysis was performed.

## 2 Methods

### 2.1 Classification criteria

This is a Class II criteria study, with retrospective sample and clinical data collection from COVID-19 patients (27). The analytical assays and the clinical data collection were developed by different researchers and physicians in double-blind studies.

### 2.2 Study design and participants

A cohort of 120 participants was recruited between March and April 2020. We included COVID-19 patients with mild (n=40) and severe (n=40) disease course and individuals without infection (control group, n=40).

All the samples were obtained for clinical purposes. Serum samples were aliquoted and stored at −80° C until analysis.

The classification of COVID-19 patients was performed according to the established clinical chart; mild disease: unilobar alveolar pneumonia, no dyspnea, FINE I-II, CURB65 0-1, arterial oxygen saturation (SatO2) >94% and/or respiration rate (RR**)** <20 rpm, no acute kidney injury (AKI), hemodynamic stability, lymphocytes >1,200, normal levels of transaminases, lactate dehydrogenase (LDH) and troponin, and D-dimer <1,000 (without previous pathology); severe disease: dyspnea, SatO_2_ <94% and RR >20 rpm, AKI, hemodynamic instability, lymphocytes <1,200, elevated transaminases, LDH and troponin, and D-dimer >1,000. Clinical, laboratory, and demographic data and comorbidities (heart disease, hypertension, obesity, diabetes mellitus, and dyslipidemia) are summarized in **Table 1**.

**Table 1:** Demographic, clinical, and analytical data from mild and severe COVID-19 patients and the control group.

	Control (n=40)	Mild (n=40)	Severe (n=40)
**Gender (Males) (n)**	23 (57.5%)	23 (57.5%)	33 (82.5%)
**Age (years, m ± SD)**	65.85 ± 3.16	68.32 ± 2.89	59.8 ± 1.47
**Bila.Pul.Infl.Infil (n)**	3 (7.5%)	1/40 (2.5%)	31/40 (77.5%)
**ICU (number) (n)**	0 (0%)	0 (0%)	40 (100%)
**Morbidity (n)**	21 (52.5%)	27 (67.5%)	19 (47.5%)
Metabolic diseases **(n)**	16 (40.0%)	21 (52.5%)	18 (45.0%)
Diabetes **(n)**	6 (15.0%)	13 (32.5%)	5 (12.5%)
Dyslipidemia **(n)**	13 (32.5%)	15 (37.5%)	8 (20.0%)
Obesity **(n)**	4 (10.0%)	6 (15.0%)	8 (20.0%)
Cardiovascular disease **(n)**	16 (40.0%)	26 (65.0%)	15 (37.5%)
Hypertension **(n)**	16 (40.0%)	25 (62.5%)	13 (32.5%)
AMI **(n)**	0 (0.0%)	0 (0.0%)	0 (0.0%)
Ictus **(n)**	2 (5.0%)	0 (0.0%)	0 (0.0%)
Cardiac insufficiency **(n)**	2 (5.0%)	0 (0.0%)	0 (0.0%)
**% Neutrophils (m)**	67.74 ± 1.68%	67.51 ± 2.43%	72.28 ± 2.16%
**Neutrophils (n/μl; m)**	5.706 ± 0.55	5.02 ± 0.66	7.416 ± 0.69
**% Lymphs (m)**	19.86 ± 1.30%	21.93 ± 1.95%	16.01 ± 1.55%
**Lymphs (n/μl; m)**	1.511 ± 0.12	1.238 ± 0.10	1.313 ± 0.12
**% Monocytes (m)**	9.01 ± 0.50%	8.52 ± 0.62%	7.71 ± 0.48%
**Monocytes (n/μl; m)**	0.678 ± 0.39	0.562 ± 0.53	0.705 ± 0.59
**% Eosinophils (m)**	2.88 ± 0.60%	1.22 ± 0.20%	3.42 ± 0.55%
**Eosinophils (n/μl; m)**	0.2171 ± 0.49	0.0877 ± 0.02	0.275 ± 0.48
**% Basophiles (m)**	0.028 ± 0.007	0.0175 ± 0.006	0.037 ± 0.008
**Basophiles (n/μl; m)**	0.51 ± 0.04%	0.51 ± 0.07%	0.59 ± 0.07%
**PT (sec; m)**	17.668 ± 4.77	13.615 ± 0.49	12.84 ± 0.18
**PT (%; (m)**	86.6 ± 3.3%	83.2 ± 3.6%	83.1 ± 2.9%
**PT INR (m)**	1.47 ± 0.39	1.15 ± 0.04	1.09 ± 0.02
**APTT (sec; m)**	34.59 ± 3.53	30.73 ± 0.53	37.52 ± 7.23
**APTT ratio (m)**	1.127 ± 0.11	0.99 ± 0.018	0.98 ± 0.026
**Fibrinogen (mg/dl; m)**	550.38 ± 23.77	563.27 ± 28.8	552.38 ± 34.72
**D-dimer (ng/dl; m)**	4708.27 ± 2081.82	1187.35 ± 162.96	4617.89 ± 906.54
**Urea (mg/dl; m)**	45.45 ± 6.17	52.63 ± 6.58	56.60 ± 6.65
**TG (mg/Dl; m)**	140.92 ± 11.39	127.53 ± 8.90	232.68 ± 23.15
**Troponin (ng/ml; m)**	0.052 ± 0.010	0.019 ± 0.010	0.103 ± 0.010
**CRP (mg/dl; m)**	4.52 ± 0.99	4.99 ± 0.95	4.21 ± 0.97
**Creatinine (mg/dl; m)**	0.986 ± 0.099	1.246 ± 0.250	0.788 ± 0.105
**Glome filtrate (ml/min; m)**	82.18 ± 4.89	76.22 ± 4.62	103.66 ± 5.14
**LDH (U/L; m)**	498.55 ± 27.81	480.35 ± 23.7	755.70 ± 45.8
**ALT (U/L; m)**	26.67 ± 3.85	30.78 ± 3.72	43.98 3.87
**AST (U/L; m)**	28.92 ± 3.85	30.78 ± 3.72	43.98 ± 3.87
**ALP (U/L; m)**	106.67 ± 9.45	92.00 ± 6.85	145.10 ± 17.36

n, number; m, mean ± standard deviation; Bila.Pul.Infl.Infil, bilateral pulmonary inflammatory infiltrates; ICU, patients admitted to the intensive care unit; AMI, acute myocardial infarction; PT, prothrombin time; INR, international normalized ratio; APTT, activated partial thromboplastin time; TG, triglycerides; glome filtrate, glomerular filtrate; LDH, lactate dehydrogenase; CRP, C-reactive protein; ALT, alanine aminotransferase; AST, aspartate aminotransferase; ALP, alkaline phosphatase.

### 2.3 Diagnostic blood tests

Blood cell counts were performed using a Beckman Coulter DXH900 hematology analyzer (Beckman Coulter^®^).

Quantification of fibrinogen, D-dimer, urea, triglycerides, troponin, C-reactive protein, creatinine, lactate dehydrogenase, alanine aminotransferase, aspartate aminotransferase, and alkaline phosphatases was carried out using a BQ AU5800 clinical chemistry analyzer (Beckman Coulter^®^) and the appropriate commercial Kits (Beckman Coulter^®^).

Fibrinogen and D-dimer levels, prothrombin time, and partial thromboplastin time were analyzed using the Coagulation analyzer ACL TOP 750 CTS (Top Diagnostic) and the corresponding commercial Kit (Top Diagnostic).

### 2.4 ELISA assay

To detect IgM and IgG antibodies to lipids we used a method published previously (25) with minimal modifications (described below). We incubated the wells with one of the following lipids: L-α-phosphatidylcholine (PC), 3-*sn*-phosphatidylethanolamine (PE), L-α-phosphatidylinositol (PI), 3-*sn*-phosphatidyl-L-serine (PS), *N*-Acyl-4-sphingenyl-1-O-phosphorylcholine (SM), 3-*O*-suphohexylceramide (SUL) and diphosphatidylglycerol, or cardiolipin (CL) (Sigma-Aldrich, St. Louis, MO, USA). Samples were diluted 1/100 in blocking solution and added to the wells in triplicate. We detected the presence of IgM or IgG to lipids in serum samples using the secondary antibodies anti human IgM (Jackson ImmunoResearch) or anti human IgG (Jackson ImmunoResearch), respectively. Positive sera were defined when the optic density (OD) was higher than the third quartile 3 (Q3) of the control group.

### 2.5 Lipidomic analysis

#### 2.5.1 Serum sample treatment

All the reagents are described in **Supplementary Data 1**. Serum samples were subjected to deproteinization and lipid extraction using a solvent mixture (methanol/chloroform/methyl *tert*-butyl ether [4:3:3, v/v/v]). Thus, samples were thawed on ice and homogenized by vortexing for 2 min. An aliquot of 40 μl of serum sample was mixed with 800 μl of the solvent mixture containing the internal standards: C17 sphinganine (2.645 μM for positive ionization mode) and deuterated palmitic acid-D31 (1.252 μM for negative ionization mode) were added. Samples were vortexed for 20 min and the pellet was removed by centrifugation at 16,100 × *g* for 10 min at 15°C. Finally, 300 μl of the supernatant was transferred to the vials with the insert Chromacol (Thermo Fisher Scientific, Madrid, Spain) for each ionization mode. Additionally, quality control samples (QC) were prepared by pooling the same aliquot (10 ul) from each sample. Furthermore, a QC for each studied group was prepared for iterative analysis and followed the same steps as the samples. Finally, blank solutions were prepared containing only H_2_O and the solvent mixture.

#### 2.5.2 RP-UHPLC-ESI-QTOF-MS sample analysis

Samples were analyzed using an Agilent 1290 Infinity II UHPLC system coupled to an Agilent 6545 quadrupole time-of-flight (QTOF) mass spectrometer. The Agilent 1290 Infinity II Multisampler system, equipped with a multiwash option, was used to uptake 1 µl for the positive ionization mode and 2 µl for the negative ionization mode of the extracted samples. The method is described in detail in **Supplementary Data 1** and in our previous publications (28). Data were processed using MassHunter Qualitative software v B.10.00 (Agilent Technologies Inc.) and MassHunter Profinder software v 10.0.2. MS/MS data sets were processed using MassHunter Lipid Annotator (Agilent Technologies Inc., Santa Clara, CA, USA) and MS-DIAL v.4 (RIKEN Center of Sustainable Resource Science, Yokahoma City, Kanagawa, Japan). To complete the lipid series, a tentative identification of lipid features was carried out based on Full Scan (MS1) data, retention time mapping (RT mapping), and the literature, using the online tool CEU Mass Mediator (CMM) (29) and the software MassHunter Qualitative v 10.0 (Agilent Technologies Inc). A final in-house library of glycerophospholipids (GPs) and sphingomyelins (SMs) was generated and used for the identification of lipid species.

### 2.6 Statistics

Clinical data were statistically compared using GraphPad Prism (version 6.0) and IBM SPSS 24 statistical packages; *p*-values <0.05 were considered statistically significant. A Mann-Whitney test was used to compare quantitative variables (age of patients, number of cells, biochemical data, and antibody levels). To analyze the percentage in the three groups, male/female, comorbidities, high levels of dimer-D (>1000) and individuals with positive serum for antibody to lipids in the different groups, we used the χ^2^ test.

To analyze the levels of IgG and IgM to lipids in each individual, we performed a heatmap protocol. Briefly, a script in R programming language was used to generate the heatmap, using the following libraries: pheatmap, RColorBrewer, and NbClust (version 3.0.1). The cluster analysis method used was “ward D2”. The index to be calculated was “Silhouette” (30).

The lipidomic analysis is described in detail in **Supplementary Data 1**. MATLAB v R2018b (The MathWorks, Maticks, MA, USA) software was used for lipidomic statistics and data normalization (31). The K-Nearest-Neighbor (K-NN) algorithm was applied to replace those data with negative values with the most probable value considering the values of their group. The algorithm support vector regression QC-SVRC-Quality Control Samples and Support Vector Regression were used for normalization.

SIMCA-P v 16.0.1 (Umetrics, Umea, Sweden) software was used for multivariate analysis (MVA). The MVA was used to reduce the dimensionality of the data (high number of lipid species per sample) to obtain a global picture of the samples. The matrix was represented in a principal component analysis (PCA-X) model for the detection of outliers that were significantly different with a confidence level >99%. Then, to obtain differences between groups, supervised models such as partial least squares-discriminant analysis (PLS-DA) and orthogonal projection on latent structures discriminant analysis (OPLS-DA) were used. The quality of these models was evaluated through the explained variance (R^2^) and prediction capacity (Q^2^) and were validated using CV-ANOVA (*p*-value ≤ 0.05).

Lipid species from MVA were also represented in a volcano plot that distributed the lipids in a combined way depending on the variable importance in projection (VIP) value and their correlation coefficient with their group, |*p*-corr|. Therefore, the selection criteria for significant metabolites in the MVA were VIP >1.0 and *p*-corr >|0.5|.

To compare the levels of each lipid species independently among the three groups, SPSS v27.0 statistical software (IBM^®^ SPSS^®^) was used to develop parametric test ANCOVA based on gender (severe COVID-19 group showed a higher percentage of males than the other groups [*p*-value 0.032]). To identify variation between groups, the percentage change was calculated as follows: (percent change = [{mean for case group – mean for control group}/mean for control group] ×100). A percentage change of >0 was interpreted as an upward trend and a percentage change of <0 as a downward trend.

## 3 Results

### 3.1 Demographic and clinical data from COVID-19 patients and the control group

Demographic, clinical, and analytical data are summarized in **Table 1**. Severe COVID-19 patients were younger than patients with mild disease (*p*=0.11). The percentage of males was higher in severe COVID-19 patients than that of mild COVID-19 patients (*p*=0.013) and controls (*p*=0.013).

There were no significant differences in the proportion of people with comorbidities (cancer, diabetes, hypertension, or respiratory disease) between the different groups.

The percentage of individuals with bilateral pulmonary inflammatory infiltrates was higher in severe COVID-19 patients than in those with mild disease (*p*<0.0001). All severe COVID-19 patients were admitted to the intensive care unit, whereas none of the patients with moderate disease were admitted.

Triglyceride levels were higher in patients with severe disease than in those with mild disease (*p*<0.0001) and the control group (*p*<0.0001). LDH levels were higher in severe cases than in the mild (*p*<0.0001) and control (*p*<0.0001) groups.

Severe cases showed higher levels of alanine aminotransferase (ALT) than mild (*p*<0.0001) and control cases (*p*<0.0001). Additionally, the severe COVID-19 group exhibited higher levels of aspartate aminotransferase (AST) than the mild (*p*=0.001) and control (*p*<0.0001) groups. However, creatinine levels were lower in patients with severe disease than in the mild (*p*<0.0001) and control (*p*=0.001) groups. No significance differences were detected in any of the parameters described above when mild COVID-19 and the control group were compared.

### 3.2 Blood counts in COVID-19 patients and the control group

The number of monocytes, lymphocytes, and basophiles was similar in the three groups (**Table 1**). COVID-19 patients with mild disease had a lower number of neutrophils than severe cases. Mild COVID-19 patients had a lower eosinophil count than severe cases and controls. No significant differences were detected between the severe cases and the control group (**Table 1**, **Supplementary Figure 1**).

### 3.3 Hemostasis analysis in COVID-19 patients and the control group

The study of hemodynamic stability showed that most of the mild and severe COVID-19 patients and control individuals had normal values for prothrombin time (PT; measured in seconds, percentage, or international normalized ratio [INR]) and activated partial thromboplastin time (aPTT; measured in seconds and ratio). By contrast, a higher percentage of severe COVID-19 patients (86.1%) showed increased levels (>1000 ng/ml) of D-dimer than the mild condition (37.5%) and control (37.8%) groups (**Supplementary Figure 2**).

### 3.4 Mild COVID-19 patients have an increased concentration of IgM to lipids

**Figure 1:**
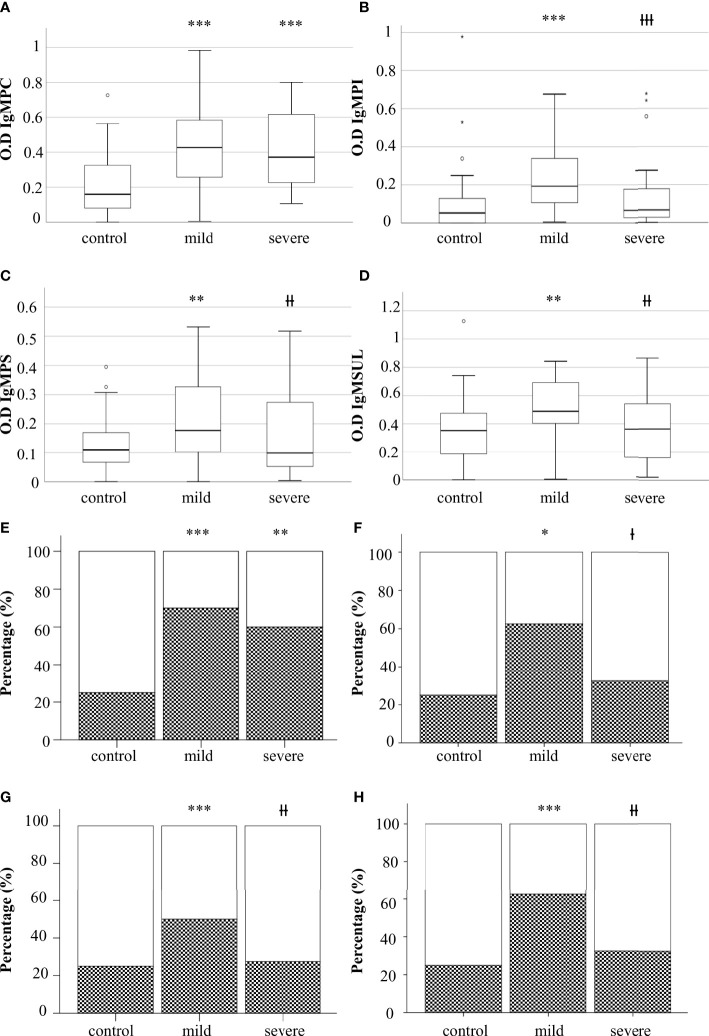
Levels of IgM to glycerophospholcholine (IgMPC) **(A)**, IgM to glycerophosphoinositol (IgMPI) **(B)**, IgM to glycerophosphoserine (IgMPS) **(C)**, and IgM to sulfatides (IgMSUL) **(D)** measured as optical density (OD). Percentage of individuals with IgM to PC **(E)**, PS **(F)**, PI **(G)**, or SUL **(H)**. Boxes represent the median of the concentration ± percentiles 25–75, and whiskers include 100% of patients. Control, control group. White bars, percentage of negatives. Squared bars, percentage of positives. Mild, COVID-19 patients with mild disease; severe, severe COVID-19 patients. *p<0.05, **p<0.01, and ***p<0.001, significantly different from the control group; Ɨp<0.05, ƗƗp<0.01, and ƗƗƗp<0.001 significantly different from the mild condition group.

We analyzed the levels of IgG and IgM to lipids in serum samples (**Table 2**). Mild and severe COVID-19 patients showed increased levels of IgM to phosphatidylcholine (IgMPC) than the control group. Mild COVID-19 patients exhibited higher levels of IgM to glycerophosphoinositol (IgMPI), glycerophosphoserine (IgMPS), and sulfatides (IgMSUL) than severe COVID-19 patients and the control group (**Figures 1A–D**).

**Table 2:** Serum levels of IgG and IgM to lipids in mild and severe COVID-19 patients and the control group.

	Control (n=40)	Mild (n=40)	Severe (n=40)
IgMPC (m ± SD)	0.219 ± 0.029	0.44 ± 0.038	0.419 ± 0.034
% positives	25.0%	70.0%	60.0%
IgMPE (m ± SD)	0.727 ± 0.06	0.662 ± 0.041	0.784 ± 0.05
% positives	25.0%	17.5%	20.0%
IgMPI (m ± SD)	0.103 ± 0.028	0.233 ± 0.026	0.128 ± 0.025
% positives	27.5%	72.5%	35.0%
IgMPS (m ± SD)	0.129 ± 0.015	0.21 ± 0.021	0.156 ± 0.022
% positives	25.0%	50.0%	27.5%
IgMSM (m ± SD)	0.26 ± 0.034	0.46 ± 0.029	0.429 ± 0.044
% positives	25.0%	62.5%	52.5%
IgMSUL (m ± SD)	0.35 ± 0.035	0.513 ± 0.035	0.374 ± 0.036
% positives	25.0%	62.5%	32.5%
IgMCL (m ± SD)	0.168 ± 0.019	0.162 ± 0.025	0.217 ± 0.034
% positives	25.0%	15.0%	30.0%
IgGPC (m ± SD)	0.287 ± 0.038	0.286 ± 0.028	0.318 ± 0.042
% positives	25.0%	25.0%	27.5%
IgGPE (m ± SD)	0.541 ± 0.034	0.514 ± 0.026	0.567 ± 0.04
% positives	22.5%	12.5%	32.5%
IgGPI (m ± SD)	0.274 ± 0.049	0.19 ± 0.028	0.199 ± 0.034
% positives	22.5%	15.0%	17.5%
IgGPS (m ± SD)	0.191 ± 0.024	0.161 ± 0.021	0.165 ± 0.025
% positives	25.5%	17.5%	15.0%
IgGSM (m ± SD)	0.278 ± 0.035	0.297 ± 0.035	0.28 ± 0.04
% positives	25.0%	30.0%	22.5%
IgGSUL (m ± SD)	0.259 ± 0.031	0.289 ± 0.034	0.261 ± 0.035
% positives	25.0%	32.5%	22.5%
IgGCL (m ± SD)	0.191 ± 0.025	0.225 ± 0.036	0.21 ± 0.034
% positives	25.0%	25.0%	17.5%

m ± SD, mean ± standard deviation; PC, phosphatidylcholine; PE, phosphatidylethanolamine; PI, phosphatidylinositol; PS, phosphatidylserine; SM, sphingomyelin; SUL, sulfatides; CL, cardiolipin.

No significant differences were detected when the levels of IgM to glycerophosphoethanolamine (IgMPE), sphingomyelin (IgMSP), or cardiolipin (IgMCL) were analyzed. Additionally, we did not detect differences between groups when we analyzed the levels of IgG to the different antigens (data not shown).

### 3.5 Antibodies to phosphatidylinositol are the main response to SARS-CoV-2

To define in detail the role of antibodies to lipids in the defense to SARS-CoV-2, we analyzed the percentage of individuals positive for IgMPC, IgMPS, IgMPI, and IgMPSUL in the three groups (**Table 2**). The presence of serum IgMPC was higher in mild and severe COVID-19 patients than in the control group. The percentage of individuals positive for IgMPC, IgMPI, IgMPS, and IgMSUL was higher in mild COVID-19 patients than in those with severe disease and in the control group (**Figures 1E–H**).

### 3.6 A high percentage of mild COVID-19 patients have IgMPI and/or IgMPS, IgMSUL, and IgMPC

To evaluate in detail the role of the immune response to lipids in the defense against SARS-CoV-2, we performed a heatmap study to analyze the presence of these immunoglobulins in each patient. In this study, the patients and lipid antibodies were grouped into clusters.

**Figure 2:**
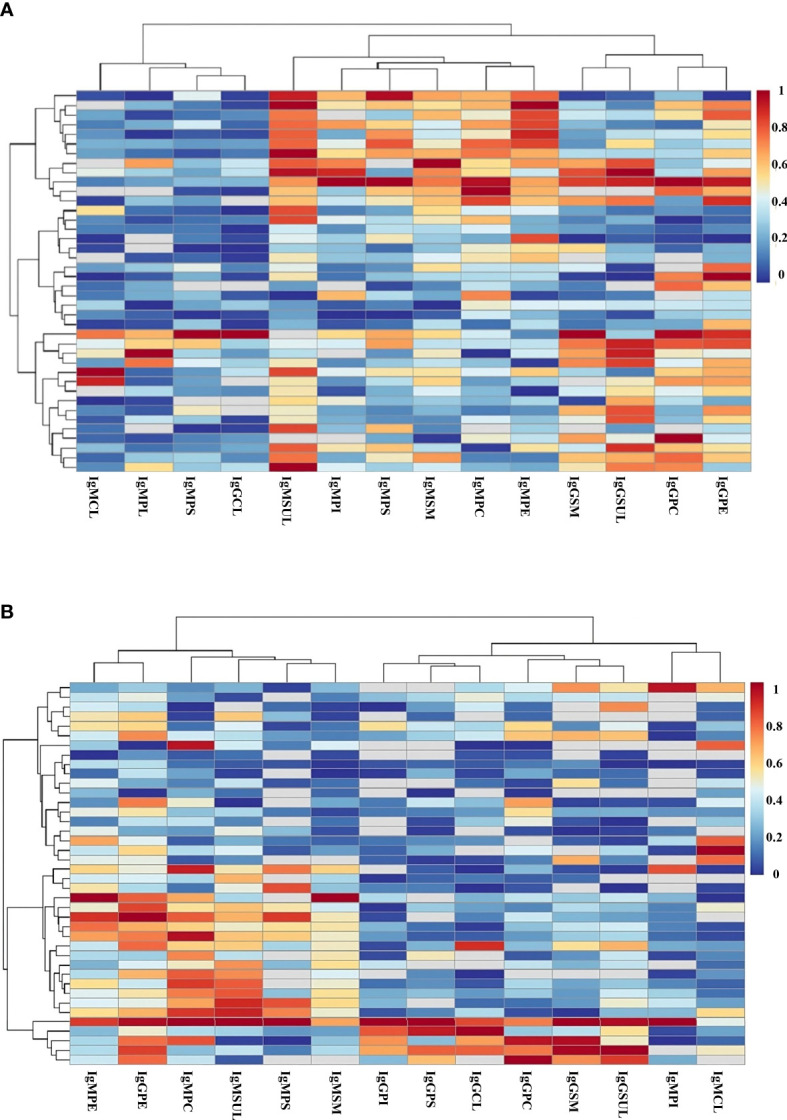
Heatmap analysis of the levels of IgG and IgM antibodies to lipids in serum samples from patients in mild **(A)** and severe **(B)** COVID-19 groups. Clustering of antibodies (y axis) and patients (x axis) considering the level of immunoglobulin to the specific lipid in each patient. Red color and blue color indicate the highest and lowest levels of antibodies to lipids, respectively. PC, phosphatidylcholine; PE, phosphatidylethanolamine; PI, phosphatidylinositol; PS, phosphatidylserine; SM, sphingomyelin; SUL, sulfatides; CL, cardiolipin.

Heatmap analysis showed that IgMPI, IgMSUL, IgMPS, and IgMPC formed a cluster in mild COVID-19 patients. In fact, 28 out of 29 patients with IgMPI also had IgM to another lipid. Moreover, we detected four patients who were negative for IgMPI but positive for IgMPC plus IgMSUL (n=3) or IgMPC plus IgMPS (n=1). In summary, 82.5% of mild COVID-19 patients had IgMPI or IgMPC plus IgMSUL or IgMPS (**Figure 2A**).

However, no clustering for IgMPI, IgMSUL, IgMPS, and IgMPC was detected in severe COVID-19 patients (**Figure 2B**).

### 3.7 Levels of antibodies to phospholipids are not related with coagulation abnormalities

A correlation between antibodies to lipids and microthrombi in COVID-19 has been observed previously, but other groups did not confirm these results (32–37). Thus, we evaluated the relationship between the presence of antibodies to lipids, using our technique and D-dimer levels.

Similar levels of IgG and IgM to lipids were detected in patients with high (>1000 ng/ml) and low concentrations of D-dimer (data not shown). Additionally, we did not detect a higher prevalence of positive sera for antibodies to lipids in COVID-19 patients with the highest levels of d-dimer than those with the lowest levels (data not shown).

### 3.8 Lipidomic analysis of COVID-19 serum samples in terms of glycerophospholipids and sphingomyelins

**Figure 3:**
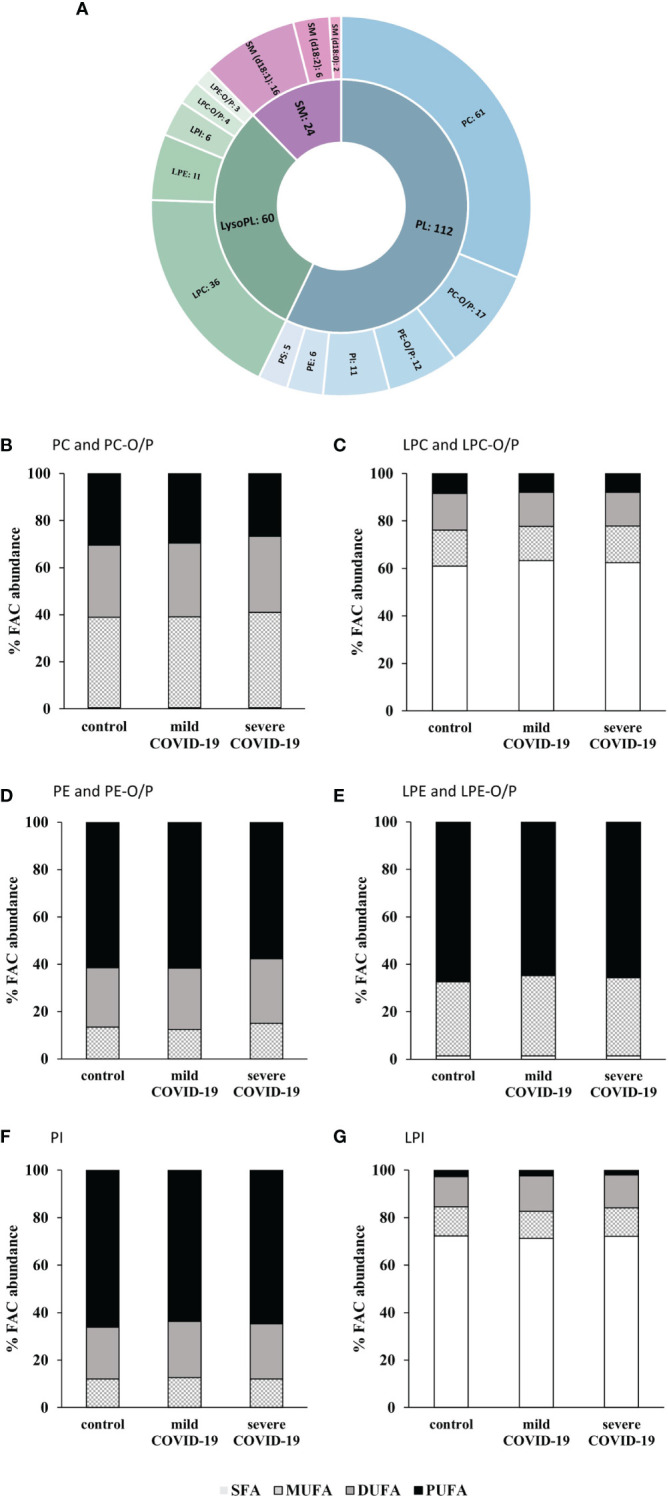
Lipidomic analysis of serum samples from COVID-19 patients and controls. **(A)** Number of glycerophospholipids (GP) and sphingomyelins (SM) identified. Glycerophosphocholines (PC), alkyl-/alkenyl-glycerophosphocholines (PC-O/P), alkyl-/alkenyl-glycerophosphoethanolamines (PE-O/P), glycophosphoinositol (PI), glycerophosphoethanolamines (PE), glycerophosphoserines (PS), lysoglycerophosphocholines (LPC), lysoglycerophosphoethanolamines (LPE), lysoglycerophosphoinositol (LPI), alkyl-/alkenyl-lysoglycerophosphocholines (LPC-O/P), alkyl-/alkenyl-lysoglycerophosphoethanolamines (LPE-O/P), and sphingomyelins (SM). Abundance percentages of fatty acyl chains (FAC) in each of the subclasses of glycerophospholipids studied. **(B)** PC and PC-O/P. **(C)** LPC and LPC-O/P. **(D)** PE and PE-O/P. **(E)** LPE and LPE-O/P. **(F)** PI. **(G)** LPI. White bars, percentage of no saturated fatty acids (SFA); squared bars, percentage of monounsaturated fatty acids (MUFA); gray bars, percentage of di-unsaturated fatty acids (DUFA); black bars, percentage of polyunsaturated fatty acids (PUFA).

Following the identification workflow described in Materials and Methods, and after performing an in-house library-assisted annotation, 196 lipid species were identified with a high confidence level. These lipid species are summarized in **Figure 3A**: 172 lipid species were glycerophospholipids (GP) [mainly phospholipids (PL) and lysophospholipids (LysoPL)], and 24 were sphingomyelins (SM) (**Table 3**).

**Table 3:** Number of identified lipids, significant lipids, and percentage of significant lipids of each lipid subclass.

Lipid subclasses	Number of lipids identified	Number of significant lipids	Significant lipids (%)
**PCs**	61	50	81.9
**PC-O/P**	17	7	41.2
**PE-O/P**	12	3	25.0
**PI**	11	10	90.9
**PEs**	6	5	83.3
**PSs**	5	4	80.0
**LPCs**	36	27	75.0
**LPEs**	11	9	81.8
**LPI**	6	5	83.3
**LPC-O/P**	4	0	0.0
**LPE-O/P**	3	1	33.3
**SMs**	24	14	58.3

PCs, glycerophosphocholines; PC-O/P, alkyl-/alkenyl-glycerophosphocholines; PE-O/P, alkyl-/alkenyl-glycerophosphoethanolamines, PI, glycerophosphoinositol, PEs, glycerophosphoethanolamines; PSs, glycerophosphoserines; LPCs, lysoglycerophosphocholines; LPEs, lysoglycerophosphoethanolamines; LPI, lysoglycerophosphoinositol; LPC-O/P, alkyl-/alkenyl-lysoglycerophosphocholines; LPE-O/P, alkyl-/alkenyl-lysoglycerophosphoethanolamines; SMs, sphingomyelins.

Once the annotation was verified, a closer inspection of the fatty acyl chain (FAC) distribution, to characterize these lipids in detail, revealed subclass-specific differences of the species described above. FACs were grouped in saturated fatty acids (SFAs), monounsaturated fatty acids (MUFAs), di-unsaturated fatty acids (DUFAs), and polyunsaturated fatty acids (PUFAs).

Glycerophosphocholines (PC) had almost no SFAs in their composition but MUFAs, DUFAs, and PUFAs were in similar proportions (**Figure 3B**). A higher concentration of SFA was observed for lysoglycerophosphocholines (LPCs) and lysoglycerophosphoinositol (LPI), (**Figure 3C, G**). PUFAs were the most abundant type of acyl chain in glycerophosphoethanolamines (PE), glycerophosphoinositol (PI), and lysoglycerophosphoethanolamines (LPE) (**Figures 3D–F**).

### 3.9 Severe COVID-19 patients have a different lipidomic profile than mild COVID-19 and control individuals

**Figure 4:**
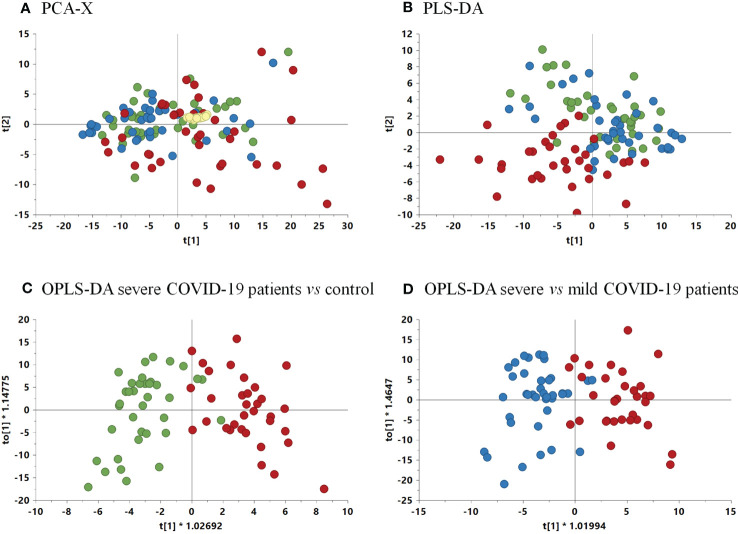
**(A)** Non-supervised PCA-X model among samples and QCs. (R^2 =^ 0.635, Q^2 =^ 0.578). **(B)** PLS-DA model score plot (R^2 =^ 0.52, Q^2 =^ 0.274) among the three groups. **(C)** OPLS-DA model comparing severe COVID-19 patients vs. control group (*p*-value 2.08 x 10^-11^) (R^2 =^ 0.549, Q^2 =^ 0.606). **(D)** OPLS-DA comparing severe vs. mild COVID-19 patients (*p*-value 5.91 x 10^-11^) (R^2 =^ 0.595, Q^2 =^ 0.535). Green, control group; blue, mild COVID-19 patients; red, severe COVID-19 patients.

To reduce the dimensionality of the data and to have a global vision of how patients behave, MVA was performed. Thus, before the comparison of the samples by MVA, quality data including data normalization were proven by the clustering of the QC samples in the PCA-X model (**Supplementary Figure 3**). In addition, for the determination of outliers, another PCA-X model was generated, leading to the elimination of those atypical values (**Figure 4A**).

For the determination of the global differences among groups, a PLS-DA model was performed. The resulting model showed a slight tendency of severe COVID-19 patients to separate from the mild condition and control groups (**Figure 4B**). This separation was confirmed by the discriminant analysis OPLS-DA models that were performed comparing paired groups, leading to the separation of severe COVID-19 vs. control and vs. mild condition groups (**Figures 4C, D**). The *p*-values of the OPLS-DA models were 2.08 × 10^-11^ and 5.91 × 10^-11^ for the comparison of severe condition *vs* controls and severe *vs* mild condition respectively (**Figures 4C, D**). Therefore, these models demonstrated a clear separation between the severe condition group and both the control and mild condition groups.

**Figure 5:**
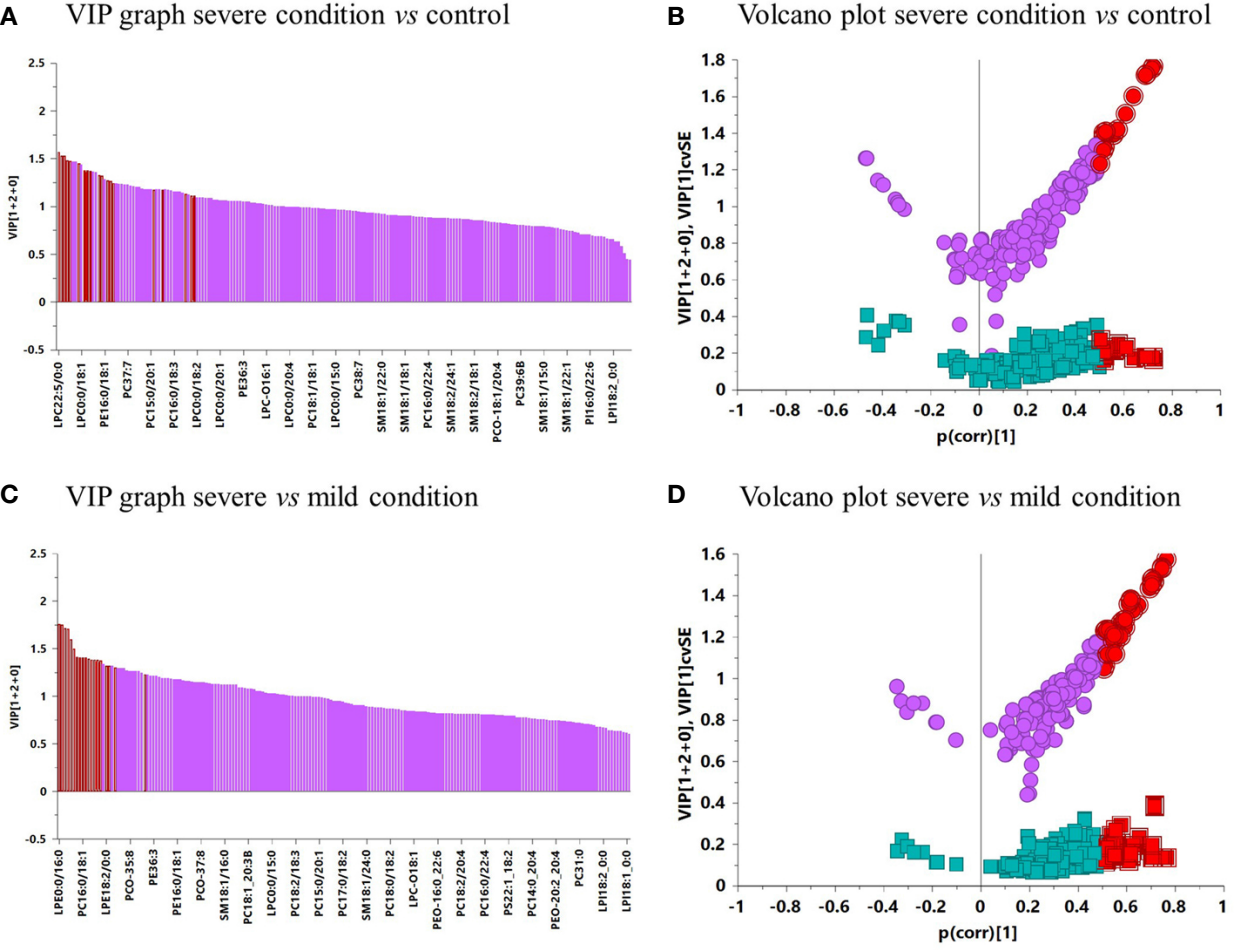
**(A)** VIP graph of severe condition vs. control. **(B)** Volcano plot of severe condition vs. control. **(C)** VIP graph of severe vs. mild condition. **(D)** Volcano plot of severe vs. mild condition. In the VIP graphs and volcano plots, lipids highlighted in red were significant in each comparison.

After the validation of the OPLS-DA models, a VIP graph and volcano plot were constructed for both comparisons to determine the significant lipids that allow differentiation between the groups studied (**Figure 5**). The results clarified 11 lipid species predicting the difference between the severe condition and the control, and 17 lipid species predicting the difference between severe and mild conditions. The main differences between both comparisons were mostly due to several species of PCs, PEs, LPCs, and LPEs.

### 3.10 Severe COVID-19 patients have higher levels of lipid species than mild and control individuals

In addition to the MVA analysis, univariate analysis (UVA) was performed to demonstrate the significance of each lipid species independently adjusted for sex, a covariate that was significant in the statistical analysis of the clinical data. From the 196 annotated GPs and SMs, 135 lipids were capable of significantly discriminating among the three groups. All the significant lipids showed higher values of estimated fold change (FC) and Cohen’s d when comparing severe COVID-19 patients with mild COVID-19 patients and the control group. However, in the comparison of mild condition vs. control, there were non-significant values. By analyzing the FC and the global tendency in each lipid subclass, we determined that mild COVID-19 patients and controls had similar levels of all the lipid subclasses; however, when comparing severe vs. mild condition and vs. controls, we found a significant increase in all the lipid subclasses studied (**Table 4**).

**Table 4:** Fold changes (FC) of the lipid species that were significant in MVA and UVA of severe COVID-19 patients vs. mild COVID-19 and control groups.

Lipid species	Fold change (FC)		
	Severe COVID-19 vs. control	Severe vs. mild COVID-19	Mild COVID-19 vs. control
PC 16:0/18:1	1.65	1.90	–
PC-O/P 16:1/18:2	1.80	1.88	–
PE 18:1/18:1	1.66	1.89	–
LPC 16:0	1.44	1.44	–
LPC 22:5	1.59	1.91	–
LPE 16:0	1.88	1.85	–
LPE 18:0	2.18	2.04	–
LPE 18:1	1.42	1.43	–
LPE-O/P 18:1	1.80	1.51	–
LPI 16:0	1.59	1.59	–

PC, glycerophosphocholines; PC-O/P, alkyl-/alkenyl-glycerophosphocholines; PE, glycerophosphoethanolamines; LPC, lysoglycerophosphocholines; LPE, lysoglycerophosphoethanolamines; LPE-O/P, alkyl-/alkenyl-lysoglycerophosphoethanolamines; LPI, lysoglycerophosphoinositol.

Finally, we identified 10 common lipid species with significant outcomes from MVA and UVA that were highly increased in severe COVID-19 patients compared with the mild condition and control groups (**Table 4**).

It is worth noting that of the significant lipid species observed, PC 16:0/18:1, alkyl-/alkenyl-glycerophosphocholines (PC-O/P) 16:1/18:2, LPC 16:0, and LPE 18:1 were the most abundant species in their corresponding subclass, while LPC 22:5, LPE 16:0, and LPE 18:0 were among the least abundant species in their corresponding subclass.

Owing to the similarities observed between mild COVID-19 patients and controls, there were no significant lipids found in any of the statistical analyses performed for this comparison.

## 4 Discussion

Antibodies to lipids, part of the so-called natural antibodies, are a main immune response to viruses (7–19); therefore, we hypothesized that they could play a major role in COVID-19. Thus, we aimed to analyze the presence of antibodies to lipids in COVID-19 patients using the most sensitive assay (25, 26).

COVID-19 patients with a mild and severe course showed an increased production of IgMPC. This was not surprising because this is the main antigen recognized by natural antibodies, and more than 50% of splenic B-lymphocytes secreting IgM are specific for this antigen (6). Moreover, the envelope of SARS-CoV-19 is enriched in phosphatidylcholine (38). Nevertheless, the similar levels of IgMPC in patients with mild and severe disease indicates that this humoral response is not strong enough to prevent the progression of the infection.

In addition, mild COVID-19 patients had higher levels of IgMPI, IgMPS, and IgMSUL than those with severe COVID-19. Indeed, the 82.5% of mild COVID-19 patients were positive for IgMPI or IgMPC plus IgMSUL or IgMPS. Heatmap analysis demonstrated that most mild COVID-19 patients showed antibodies to more than one lipid. However, we did not observe either the high prevalence or the coexistence of antibodies to these lipids in severe COVID-19 patients.

Our data differ from previous reports suggesting a pathological role of antibodies to lipids. These studies indicate a correlation between the presence of antiphospholipid antibodies (32, 39–44) and disseminated microthrombi, which have been observed in almost 30% of patients with worsening pneumonia and in some fatal cases (32, 33).

On the contrary, other authors did not detect this correlation (34–37), or detected antiphospholipid antibodies in patients with a moderate disease (45). In this line, we observed that severe COVID-19 patients had higher levels of D-dimer and fibrinogen, markers of coagulopathy, than those with mild COVID-19, as described previously (41, 46). Nevertheless, we did not detect a relationship between the presence of antibodies to any of the lipids analyzed and coagulopathy markers.

Our goal was to use the most sensitive technique for the detection of antibodies to lipids (25, 26). We analyzed the serum antibody reactivity to different lipids (PC, PE, PI, PS, ESF, and SUL) from those previously investigated (cardiolipin and β2-glycoprotein I (β2GPI)) (47).

However, our data correlate with previous results indicating that antibodies to PI, PS, PC, PE, and cardiolipin are neutralizing (48–52). Phosphatidylinositol is the main component of the envelop of SARS-CoV-2 (38). The downregulation of IgMPI and IgMSUL in severe COVID-19 could diminish the control of the replication of SARS-CoV-2. Regarding this, it was previously described a higher viral burden in those individuals with severe disease (53). Most of the severe patients analyzed in our cohort were men, corroborating previous results (54), and they had lower levels of natural antibodies than females (55). Another function of antibodies to lipids is to eliminate dying cells (6), because during apoptosis phosphatidylserine is oxidized and translocated to the outer leaflet of the lipid bilayer (56). PS is not a main antigen of the viral envelope (38); therefore, these data suggest that IgMPS could participate in the clearance of SARS-CoV-2-induced dying cells rather than the elimination of the virus.

Defeating the virus or eliminating dying cell antibodies to lipids regulate the inflammatory response (6). Corroborating previous results, we observed a higher number of neutrophils (53) and eosinophils in severe COVID-19 patients.

Viruses, including the Coranoviridae family, hijack the lipid metabolism of the host cell to create the most favorable lipid micro-environment for their replication (21).

We observed that severe COVID-19 patients showed increased levels of glycerophospholipids (PCs, PC-O/P, LPCs, LPC-O/P, PEs, PE-O/P, LPEs, and LPE-O/P) and sphingomyelins, corroborating previous results (57–61). The UVA and MVA results identified 11 of the 196 lipid species that were significant in both comparisons, suggesting that they may be key metabolites in disease progression. Two of the significant metabolites found in severe cases, PC-O/P 16:1/18:2 (PC-O/P 34:3) and LPC 16:0, have previously been described as potential predictors of COVID-19 disease severity (59, 62). In this regard, other groups demonstrated that the levels of other lipid species (PC O-34:3, LPC 18:0, LPC 20:1, LPC O-16:0, LPC O-16:1, and LPC O-18:1) could be predictors of severity status (59).

On the contrary, some published studies observed a significant decrease in PCs, PC-O/P, LPCs, and LPC-O/P (59, 63), whereas others indicated a decrease in PCs but an increase in LPCs (57, 64, 65). This controversy is not surprising, as a study that evaluated the role of lipid metabolites in acute respiratory distress syndrome demonstrated a decrease in PCs as the disease progressed, high levels of PEs in each phase of the disease, and an increase in LPCs in the late phase (66). The samples we analyzed were, in most cases, obtained 1 week after the onset of the symptoms.

LPC is highly cytotoxic to type II pneumocytes (67) and induces the disruption of the alveolar epithelial barrier (68). Moreover, LPC induces gene transcription in endothelial cells, smooth muscle, and fibroblast, and upregulates the expression of growth factor (69) and monocyte chemoattractant protein-1 (70). Additionally, LPC increases oxidative stress in hepatocytes (71) and endothelial cells (72) and induces vascular and hepatic dysfunction, which are typical mechanisms in severe COVID-19 disease (73). LPC promotes the secretion of the proinflammatory cytokines IL-1, IL-6, and TNF-α (74). This lipid regulates the immune response, activating B cells (75), promoting chemotaxis (76) and macrophage activation (77). Proinflammatory cytokines and infiltrating macrophages are hallmarks of patients with severe disease (78).

The modulation of the host cell lipidic metabolism by the Coronaviridae family is mediated *via* cytosolic phospholipase A2α enzyme (22). The latter cleaves phospholipids to form lysoglycerophospholipids and arachidonic acid, which are vital for the formation of the viral membrane; thus, this enzyme could be a potential therapeutic target (21).

In summary, our work draws attention to the high prevalence of IgM to lipids in mild COVID-19 patients but not in severe COVID-19 patients. To our knowledge, this is the first time these groups have been so clearly differentiated, and this differentiation indicates that these immunoglobulins are the highest sensitivity and specificity prognosis biomarkers of the disease. Secondly, although we realize we did not demonstrate the neutralizing activity of IgM to lipids, it was previously demonstrated by other groups (48–52). All these data suggest that IgMPI, IgMPC and IgMSUL could be a new therapeutic tool for those patients with a severe course. Regarding this, patients with low levels of antibodies to lipids showed an increased proinflammatory lipidomic profile. The regulation of this pathway is another target for developing new therapeutic approaches for patients with severe disease.

## Data availability statement

The original contributions presented in the study are included in the article/**Supplementary Material**. Further inquiries can be directed to the corresponding authors.

## Ethics statement

All the protocols were approved by the Bioethics Committee of Hospital Clínico San Carlos (Madrid, Spain) and Hospital Universitario HM Madrid. To obtain the samples, all the patients gave the verbal and written informed consent. The patients/participants provided their written informed consent to participate in this study.

## Author contributions

UM, MS, EE, and AG: study conception and design; IP, IM, SM, VA-H, and AV: acquisition and analysis of data. IP, MS, UM, EE, IS-V, AG, SM, AV, and IM: drafting a significant portion of the manuscript or figures and tables. JV, AR, and JC: provision of samples and clinical data revision. All authors contributed to the article and approved the submitted version.
