# Supplementary material for: Deficiency in the production of antibodies to lipids correlates with increased lipid metabolism in severe COVID-19 patients
Source: Front Immunol. 2023 Jun 23;14:1188786. doi: 10.3389/fimmu.2023.1188786 (PMC10327431; doi:10.3389/fimmu.2023.1188786)
Supplement: Supplementary file 1 [file DataSheet_1.docx]

**Supplementary Data 1. Lipidomic analysis**

**1. Reagents.**

Reverse osmosis ultrapure water, (Milli‐Qplus185 system; Millipore, Billerica, MA, USA); LC-MS grade methanol (MeOH); acetonitrile (ACN), and isopropanol (IPA) (Fisher Scientific; Pennsylvania, United States); HPLC grade methyl *tert*-butyl ether (MTBE), ammonium fluoride (NH_4_F) (ACS reagent, ≥98%) and the internal standards C17 sphinganine and deuterated palmitic acid D31 (Sigma‐Aldrich, Steinheim, Germany); analytical grade ammonia solution (28%, GPR RECTAPUR®) and acetic acid glacial (VWR Chemicals, Pennsylvania, United States).

**2.** **RP-UHPLC-ESI-QTOF-MS sample analysis.**

MassHunter Qualitative software v B.10.00 (Agilent Technologies Inc.) was used to generate Quality Charts, overlays of the equipment pressure, and the Base Peak Chromatograms (BPCs) of the QC samples to verify system mass accuracy, data quality, and the reproducibility of the QC samples.

Then raw data were aligned and processed with Agilent MassHunter Profinder software v 10.0.2. An untargeted analysis was performed; the datasets were extracted by using the Batch Recursive Feature Extraction (RFE) workflow that comprises two steps: Batch Molecular Feature Extraction (MFE) and Batch Find by Ion Feature extraction (FbI). MFE algorithm performs chromatographic deconvolution and (i) aligns the features across the selected sample files using mass and retention time (RT), (ii) removes unwanted information and (iii) creates a list of possible components. In addition, MFE also has the goal of detecting coeluting adducts of the same features, being the adducts selected: [M+H]^+^, [M+Na]^+^, [M+K]^+^, [M+NH_4_]^+^ and [M+C_2_H_6_N_2_+H]^+^ for LC-MS positive ionization; [M-H]^−^, [M+CH_3_COOH-H]^−^, and [M+Cl]^−^ for LC-MS negative ionization mode^33^.

For lipid annotation: The MS/MS iterative data obtained, from both positive and negative ionization modes, was processed using a combination of two independent software annotation tools: MassHunter Lipid Annotator (Agilent Technologies Inc., Santa Clara, CA, USA) and MS-DIAL v.4 (RIKEN Center of Sustainable Resource Science, Yokahoma City, Kanagawa, Japan). Lipid Annotator^34^ and MS-DIAL^35^ do a spectral matching annotation with the predicted spectral libraries. After merging into a single list, the annotation given by the two software and the MS/MS spectra were manually inspected to avoid false positives due to possible interferences between the different subclasses of lipids, due to the large presence of isobars and isomers, which suppose a great difficulty when performing reliable structural characterization.

In order to complete the lipid series, a tentative identification of lipid features was done, based on the Full Scan (MS1) data, Retention time mapping (RT mapping), and literature, using the online tool CEU Mass Mediator (CMM)^36^ and the software MassHunter Qualitative v 10.0 (Agilent Technologies Inc). A final in-house library of glycerophospholipids (GP) and sphingomyelins (SM) was generated containing molecular formula, monoisotopic mass, and RT. The nomenclature used in this article for the lipid species reported follows the recently updated IUPAC rules^37^.

The in-house library was used to perform data mining by the Batch Targeted Feature Extraction (TFE) in which the algorithm extracts features from the data using a process called Find Compounds by Formula. The resulting list was imported to an Excel file with all area data of molecular features extracted and treated before the statistical analysis. Blank signals were subtracted from all samples to remove impurities due to column cleaning or possible contaminants. To do this, the mean of the areas of the initial blanks was calculated and subtracted from the areas of each sample.

Data quality was also assessed by maintaining only those signals that, in the QC samples, had a coefficient of variation (CV) below 30% and that were present in at least 75% of all the samples under study. The Total Useful Signal (TUS) was also calculated and represented to discriminate those samples that give a low signal compared to the rest of the samples.

The QCs were injected continuously at the beginning of the analysis until system equilibration, then, QCs were injected at regular intervals – every 6 randomized samples - to assess stability, reproducibility, and performance of the system together for its use in the correction of any signal deviation within the analytical sequence. Two blanks were injected at the beginning and end of the sequence to subtract the background signals.

**3. Statistics.**

Data obtained from lipidomic studies were analyzed as described next. The filtered matrix obtained in the previous step was processed by MATLAB v R2018b (The MathWorks, Maticks, MA, USA) for normalization^39^. Before that, for those data with negative values, the K-Nearest-Neighbor (K-NN) algorithm was applied, to replace them with the most probable value considering the values of their group. For normalization, correction was done based on the data contained in the QC samples. The algorithm support vector regression (QC-SVRC-Quality Control Samples and Support Vector Regression) was used to correct the areas of each sample, using as references the areas of the QC samples. After normalization, for those data with negative values, the K-Nearest-Neighbor (K-NN) algorithm was applied, to replace them with the most probable value considering the values of their group.

The data matrix obtained was processed by SIMCA-P v 16.0.1 (Umetrics, Umea, Sweden) for multivariant analysis (MVA) and by IBM SPSS statistics v 27.0 for univariant analysis (UVA). The MVA was performed by using the software SIMCA-P v 16.0.1. Prior to analysis, data were tested using different scales: UV (autoscaling), Pareto, and centroid. Data were finally scaled using UV since it was the best one that grouped the QCs and distinguished the groups of study. The matrix was represented in a principal component analysis model (PCA-X) for the detection of outliers that were significantly different with a confidence level >99%, sample trends, analytical variability, and reduction of the dimensionality of the data. Afterwards, supervised models such as partial least squares-discriminant analysis (PLS-DA) and orthogonal projection on latent structures discriminant analysis (OPLS-DA) were performed. The quality of the constructed models was evaluated through the explained variance (R^2^) and predicted variance (Q^2^). The models were validated by CV-ANOVA (*p*-value ≤ 0.05). Outliers were eliminated based on Hotelling’s T2Range line plot with a confidence interval >99%. In addition, after obtaining the OPLS-DA model, the variable importance in projection (VIP) values of each lipid were represented as a descending column chart with confidence intervals derived from the Jackknife method. The metabolites were represented in a volcano plot that distributes the metabolites in a combined way depending on the importance of each VIP value and their correlation coefficient with their group, |*p*-corr|. Therefore, the selection criteria for significant metabolites in the MVA were VIP >1.0 and *p*-corr >|0.5|.

For the UVA, the parametric test ANCOVA (Analysis of Covariance) (IBM SPSS statistics v 27.0) was adjusted by gender, since it was significant in the statistical analysis of the clinical data (*p*-value 0.032). ANCOVA test was selected due to the presence of three study groups with a significant covariable. The selection criteria for significant lipids were set in a corrected p-value by false discovery rate (FDR) (*q*-value ≤0.05) and with a Cohen’s d >0.8. To identify variation in between groups, percent change (% change) was calculated as follows (percent change = ((mean for case group – mean for control group)/mean for control group) *100). A % change >0 was interpreted as an upward trend and a % change <0 as a downward trend.
