# Supplementary material for: Deficiency in the production of antibodies to lipids correlates with increased lipid metabolism in severe COVID-19 patients
Source: Front Immunol. 2023 Jun 23;14:1188786. doi: 10.3389/fimmu.2023.1188786 (PMC10327431; doi:10.3389/fimmu.2023.1188786)
Supplement: Supplementary file 2 [file DataSheet_2.docx]

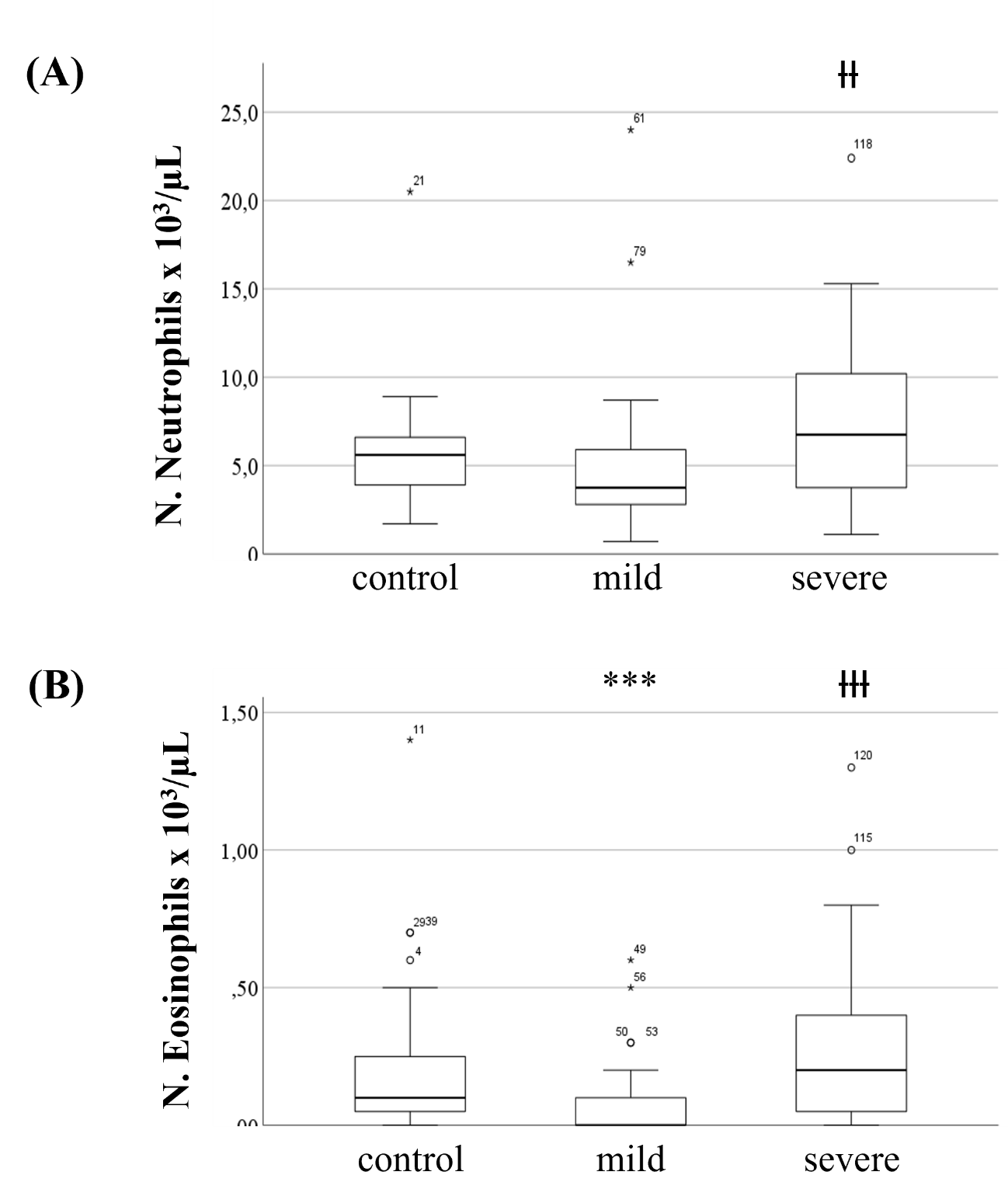


**Supplementary Figure 1.** Analysis of the number (N) of neutrophils (A), and eosinophils (B) in COVID-19 patients and the control group. Boxes represent the median of the number ± percentiles 25–75, and whiskers include 100% of the patients. Control: control group. Mild: mild COVID-19 patients. Severe: severe COVID-19 patients. ***p<0.001 significantly different from control group, ƗƗp<0.01, ƗƗƗp<0.001 significantly different


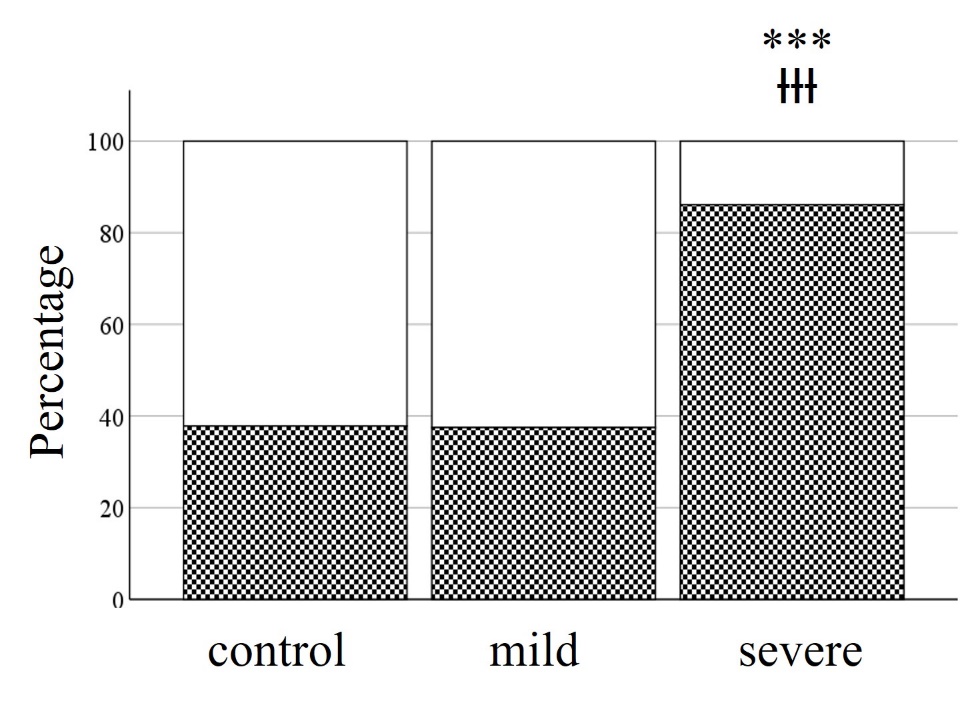


**Supplementary Figure 2.** Percentage of patients with high levels (>1,000 ng/ml) of d-dimer. White bars: percentage of patients with levels of d-dimer lower than 1,000 ng/ml. Squared bars: percentage of patients with levels of d-dimer higher than 1,000 ng/ml. Mild: COVID-19 patients with mild. Severe: severe COVID-19 patients. ***p<0.001 significantly different from the control group, ƗƗƗp<0.001 significantly different from the mild condition group.

**Supplementary Figure 3.** **(A)** Total Useful Signal (TUS) values of the QC and study samples following the injection order after normalization. **(B)** PCA-X model generated from the analysis of the QC and study samples after normalization. QC (orange), study samples (gray).
